# DFB-PPGSQ: Dynamic Forward-Private and Epochal Backward-Private Graph Similarity Matching Query over Encrypted Sensor Graph Databases

**DOI:** 10.3390/s26154804

**Published:** 2026-07-28

**Authors:** Huiying Hou, Yucong Ma, Zisu Zhao, Xinrui Ge

**Affiliations:** 1 The Key Laboratory of Grain Information Processing and Control, Henan University of Technology, Zhengzhou 450001, China; 2 The School of Artificial Intelligence and Big Data, Henan University of Technology, Zhengzhou 450001, China; ycma@stu.haut.edu.cn (Y.M.); 251210200819@stu.haut.edu.cn (Z.Z.); 3 Henan Province Key Laboratory of Cyberspace Situation Awareness, Zhengzhou Institute of Information Science and Technology, Zhengzhou 450001, China; 4 School of Computer Science and Technology, Qingdao University, Qingdao 266071, China; xinruige@qdu.edu.cn

**Keywords:** encrypted graph database, graph similarity matching, structured encryption, searchable encryption, forward privacy, epochal backward privacy, dynamic graph update, IoT security, sensor cloud

## Abstract

In applications pertaining to sensor network, the Internet of Things, industrial monitoring, and cyber–physical security, graphs are increasingly being outsourced to clouds, where similarity search should be supported without exposing graph content, query graphs, update contents, or database evolution. Existing privacy-preserving graph similarity schemes mainly target static encrypted databases, and as such struggle to handle insertions, deletions, label updates, and long-running index maintenance. This paper proposes DFB-PPGSQ, a dynamic forward-private and epochal backward-private graph similarity matching scheme that moves branch-based lower-bound filtering into a structured encryption framework. DFB-PPGSQ uses epoch-local feature tokens, per-record occurrence handles, one-time update labels, update buffers, deletion tombstones, and shuffle-based branch–tree re-randomization to preserve pruning efficiency while making same-epoch tombstone, traversal, size, timing, and refresh leakage explicit. We formalize the system model, leakage functions, algorithms, and security interpretation, then implement a reproducible Python prototype with HMAC-SHA256 token generation and multi-profile dynamic sensor-topology workloads. Across five random seeds, DFB-PPGSQ keeps server-side filtering latency close to the static branch–tree baseline (46.60 ms versus 44.72 ms at 4000 graphs), avoids immediate full-rebuild updates (0.091 ms insertion and 0.092 ms label update), and keeps metadata-assisted cross-epoch token linkage below 5.6% attack success after refresh in additional industrial and campus IoT stress workloads. Storage, communication, exact GED refinement, side-channel hardening, and verifiable-result protection are treated as deployment costs and limitations rather than being included in the headline latency.

## 1. Introduction

Graphs provide a natural data structure for describing entities and their relationships. In sensor networks, vertices can represent deployed sensors and edges can describe communication links. In Industrial IoT contexts, vertices can denote controllers, actuators, events, or data streams, while edges can express operational dependencies. In cyber–physical security, a graph may capture provenance paths, alert correlations, or lateral movement traces. Such graph data are valuable, but are also sensitive. Sensor applications are often resource-constrained and security-sensitive, meaning that privacy mechanisms must remain lightweight enough for practical IoT deployments [1].

Cloud computing provides an attractive platform for storing and querying large graph databases. A client can outsource the graph database and obtain scalable computation. However, the cloud and the client usually belong to different trust domains; the cloud can be honest in executing protocols, but curious about graph structures, labels, queries, and query results. Therefore, graph data are often encrypted before outsourcing. A meaningful encrypted graph service should still support useful operations such as graph similarity matching queries.

Graph similarity matching retrieves data graphs for which the distance to a query graph is no larger than a user-specified threshold, a more tolerant approach than exact graph matching. This tolerance is necessary in practical sensing and monitoring applications where data collection is noisy, device states are incomplete, labels can change over time, and inter-sensor dependencies may drift in dynamic environments [2]. Where a strict exact match might miss useful results, a similarity query can recover them.

Recent privacy-preserving graph similarity matching query schemes have made important progress. PGSim and PrigSim protect graph similarity search over encrypted data by combining graph filters and secure query processing [3,4]. The recent PPGSQ scheme improves efficiency by adopting a branch-based lower bound of edit distance and a branch–tree index [5]. Its core idea is to encode each local neighborhood as a branch consisting of a center vertex, incident edge labels, and neighboring vertex labels. Since a single graph edit operation affects a limited number of branches, branch-based filtering gives a tighter lower bound than a global label filter and can prune more mismatched data graphs.

However, current PPGSQ-style systems are primarily static. This is a narrow assumption for real cloud graph services. Sensor cloud databases change continuously; the database evolves as new devices join, old devices disappear, labels are corrected, abnormal events are reclassified, and communication edges are removed.

This dynamic setting changes the security problem. If encrypted branch tags remain stable, then the cloud can link updates to previous queries. Deleted graphs can leave stale index traces, future queries may reveal information about removed objects, and label updates implemented as delete-and-insert operations without privacy control require the cloud to infer which branch features have changed. In long-running services, even small leakages accumulate. Therefore, the resulting challenge involves not just computational efficiency but temporal privacy.

This paper asks the following question:

Can we support efficient graph similarity matching over encrypted graph databases while allowing insertions, deletions, label updates, and periodic index re-randomization with forward privacy and epochal delay-bounded backward privacy?

We answer this question by proposing DFB-PPGSQ. The scheme keeps the useful branch-based filtering principle but redesigns the encrypted index as a dynamic structured encryption object. The index evolves across epochs. Update tokens are one-time and unlinkable across time. Deleted records are represented by bounded tombstone information, and are removed or re-randomized during epoch compaction. The branch–tree index is preserved for efficient pruning, but its labels and counters are refreshed by epoch keys. The design does not claim volume hiding, result size hiding, oblivious traversal, or strong hidden-deletion backward privacy; instead, it states the leakage profile explicitly and evaluates the cost of reducing stable cross-epoch token equality.

The main contributions of this paper are summarized as follows:

1. We formulate dynamic forward-private and epochal backward-private PPGSQ. We extend the static graph similarity matching query model to support graph insertion, graph deletion, vertex/edge label updating, and periodic re-randomization. We define the syntax, threat model, leakage functions, and privacy goals for dynamic encrypted graph similarity services.
2. We design a dynamic branch-based encrypted index. DFB-PPGSQ constructs epoch-local feature tokens, per-record occurrence handles, encrypted branch-count vectors, update buffers, deletion tombstones, and a re-randomizable branch–tree index. The construction supports efficient filtering without forcing the cloud to traverse the whole database after each update.
3. We integrate forward privacy and epochal backward privacy with graph filtering. Search tokens generated before an update cannot be used to identify future inserted or updated branch features. Future queries reveal only bounded information about deleted graphs. Periodic re-randomization further reduces long-term linkability of branch identifiers and tree nodes.
4. We provide a reproducible prototype evaluation. We implement the protocol-relevant operations of DFB-PPGSQ in Python, including branch extraction, HMAC-based token generation, update buffering, tombstone filtering, epoch refresh, and generated dynamic sensor topology workloads. The evaluation compares DFB-PPGSQ with several baselines: static scan, static branch–tree, feature-only dynamic SSE, rebuild-after-update, and no re-randomization.

The remainder of this paper is organized as follows: Section 2 reviews related work; Section 3 presents the system model, threat model, notation, and security definitions; Section 4 introduces preliminaries; Section 5 gives the detailed construction of DFB-PPGSQ; Section 6 provides the security analysis; Section 7 presents the prototype implementation and experimental results; finally, Section 8 concludes the paper.

## 2. Related Work

### 2.1. Privacy-Preserving Graph Similarity Query

A graph similarity query is a fundamental graph database operation. Given a query graph and an edit distance threshold, the server retrieves data graphs that approximately match the query. Graph edit distance has a long history in attributed graph matching and pattern recognition [6,7]. Since exact graph edit distance computation is expensive, practical systems usually follow a filtering-and-refinement framework [8,9,10,11]. A lower bound is first computed to prune impossible candidates. The remaining graphs are then refined by a more expensive distance computation.

Encrypted graph similarity querying brings an additional constraint in which the cloud should not see plaintext labels, graph structures, or query graphs. PGSim uses pivot-based filtering to support privacy-preserving graph similarity queries over encrypted outsourced data [3]. PrigSim improves the service model and considers graph similarity search in cloud settings [4]. The recent PPGSQ design replaces heavy pivot computations with branch-based lower-bound filtering and a secure branch–tree index, which improves pruning ability and query efficiency [5].

These schemes are important foundations, yet they mainly assume that the encrypted graph database is static after setup. In practice, sensor and IoT graphs are dynamic. A static index must be rebuilt after each update or must accept privacy leakage caused by stable encrypted identifiers, neither of which provides a satisfying solution.

### 2.2. Dynamic Searchable Symmetric Encryption

Searchable encryption allows a client to outsource encrypted data while retaining search capability [12,13,14,15]; later systems provided improved scalability and expressive queries [16]. Dynamic searchable symmetric encryption further supports document insertion and deletion [17,18,19,20]. Forward privacy, introduced to prevent newly added documents from being linked to previous search tokens, has become a standard requirement for dynamic encrypted search [21]. Backward privacy limits what future searches reveal about deleted documents [22]. Stronger backward privacy notions reduce leakage from deleted records, but often require higher update cost, server-side cleanup, or more complex key evolution.

The DFB-PPGSQ problem is related to dynamic searchable symmetric encryption, but is not a direct keyword search problem. A graph similarity query touches many branch features, computes a lower bound, and traverses a tree-shaped index. Insertions, deletions, and label updates modify structured objects rather than independent documents. Therefore, dynamic searchable encryption ideas must be adapted to graph features, branch counts, candidate filtering, and tree pruning.

### 2.3. Secure Graph Query Processing

Secure graph query processing covers shortest path queries, reachability queries, subgraph matching, and graph analytics over encrypted graphs. Privacy-preserving encrypted graph query processing was explored by Cao et al. [23]. GRECS and later graph encryption schemes show that specialized encrypted graph indexes can support useful graph operations [24]. GraphShield considers dynamic secure graph queries with forward privacy [25]. The recent PathGES improves secure shortest-path query processing [26]. These systems demonstrate the value of graph-specific encrypted indexes.

Recent work on privacy-preserving multi-user graph intersection in cloud-assisted IoT published in Sensors further shows that graph operations over outsourced wireless/IoT data are becoming a concrete sensor cloud requirement [27]. However, shortest path querying, graph intersection, and graph similarity matching expose different computation patterns. A similarity query compares a query graph with many candidate graphs under an edit distance threshold, which requires feature-level counting, lower-bound computation, and pruning. DFB-PPGSQ targets this specific operation.

### 2.4. Leakage Attacks and Motivation for Re-Randomization

Encrypted search systems often leak access patterns, search patterns, result sizes, update sizes, and sometimes order information. Leakage abuse and inference attacks show that such information can be exploited with auxiliary knowledge [28,29,30,31]. Order-revealing encryption and deterministic encrypted identifiers may further expose ordering or equality relations [32]. For static encrypted graph indexes, repeated queries may reveal stable branch or node correlations.

Dynamic graph services intensify this risk. The same branch feature may appear across many epochs, while a deleted graph may still influence future result sizes. A label update may reveal a before-and-after relationship. Therefore, DFB-PPGSQ adds periodic re-randomization, as shown in Table 1. This does not eliminate all leakage, but does reduce persistent linkability while giving the data owner a tunable mechanism for limiting leakage accumulation. The refresh operation itself also leaks migration size, bucketed record sizes, refreshed tree shape, and timing; these observables are treated as part of the re-randomization leakage rather than as hidden quantities.

## 3. Problem Formulation

### 3.1. System Model

The system model contains two primary entities: the client and the cloud. It can also include authorized users in a multi-user deployment, but this paper focuses on the single-client setting first. This scope matches the basic SSE model and is intentionally narrower than multi-owner, multi-gateway, and multi-policy sensor cloud deployments. Extending the construction to those deployment models requires additional authorization, revocation, and policy enforcement layers, and is left as future work.

#### 3.1.1. Client

The client owns a dynamic graph database. They extract graph features, generates cryptographic keys, encrypts graph records, constructs secure indexes, and issues update/query tokens. The client is trusted, and may be a sensor data owner, IoT service operator, or security monitoring platform.

#### 3.1.2. Cloud

The cloud stores encrypted graphs and secure indexes, receives update tokens and query tokens from the client, executes update, filtering, pruning, and candidate retrieval procedures on encrypted data, and returns encrypted candidate graphs to the client.

**Figure 1:**
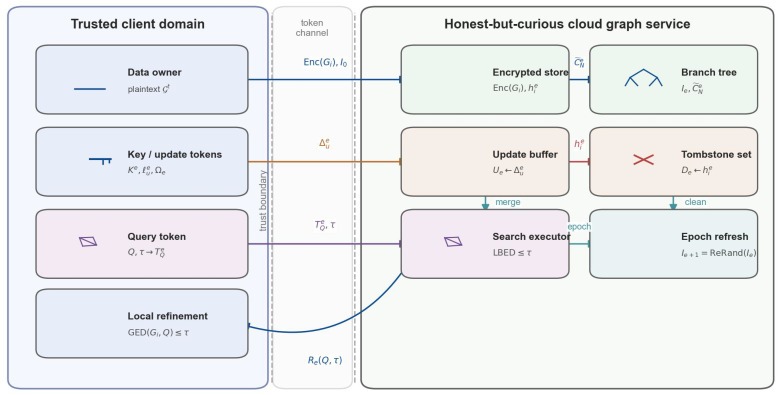
System model of DFB-PPGSQ. The figure separates the trusted client domain from the honest-but-curious cloud graph service and shows the the setup, update, query, results returning, and epoch refreshing flows.

Figure 1 summarizes the system-level interaction. The trusted client keeps plaintext graphs, master keys, and workload semantics locally, uploading only encrypted graph records and protected index material. Later, the client sends update tokens, query tokens, and epoch re-randomization material to the cloud. The cloud is honest-but-curious. It stores the encrypted graph database, branch–tree index, update buffer, tombstone set, and re-randomization state while performing token-authorized filtering and returning encrypted candidates for local decryption and optional refinement.

The dynamic graph database at logical time *t* is denoted by

(1) $$G^{t}=\left\{G_{1}^{t},G_{2}^{t},\ldots ,G_{N_{t}}^{t}\right\}.$$

Each graph $G=(V,E,L_{V},L_{E})$ contains a vertex set *V*, edge set *E*, vertex label function $L_{V}$, and edge label function $L_{E}$. A query graph is denoted by *Q*. The query threshold is denoted by τ. The query result consists of encrypted graphs for which the graph edit distance to *Q* is no larger than τ.

DFB-PPGSQ supports the following operations:

- **Insert**: Adds a new graph *G* to the encrypted database.
- **Delete**: Removes an existing graph $G_{i}$ from the active database.
- **UpdateLabel**: Modifies one or more vertex labels or edge labels in an active graph.
- **Search**: Retrieves encrypted candidate graphs that satisfy the similarity threshold.
- **ReRand**: Re-randomizes feature tokens, occurrence handles, tree nodes, counters, and active-record handles at an epoch boundary.

Topology-changing updates such as edge insertion, edge deletion, or local device neighborhood changes can be represented by recomputing the affected one-hop branches and issuing the same branch-delta update when the affected local neighborhood is available to the client. If a deployment cannot identify the local affected set, the conservative fallback is delete-and-insert, which preserves correctness but increases update cost and weakens continuity. The prototype evaluation focuses on whole-graph insertion/deletion and label updates; explicit edge update engineering is a limitation of the current implementation.

### 3.2. Threat Model

The cloud is honest-but-curious. It follows the protocol, but attempts to infer information from encrypted graphs, secure indexes, update tokens, query tokens, search traces, access patterns, result sizes, tree traversal paths, and timing metadata. This is the same broad server model used by many searchable encryption systems.

The client is trusted. It generates keys correctly and does not leak plaintext graph data. Network channels between the client and authorized users can be protected by standard authenticated encryption. Denial-of-service attacks, malicious result omission, collusion between users, and side-channel leakage from implementation details are outside the main model. Accordingly, the present security analysis should be read as honest-but-curious leakage-based security, not as a guarantee of resistance to malicious server, verifiable computation, or side-channel attacks.

The cloud may possess auxiliary knowledge, and may know public graph distributions, common sensor labels, partial plaintext graphs, or historical workload statistics. This knowledge makes leakage control more important.

Side-channel attacks, including timing, power, and electromagnetic leakage, are outside the current threat model. The honest-but-curious adversary is assumed to observe only the protocol messages and the leakage functions defined in Section 6.1, not physical side channels.

### 3.3. Notation

Table 2 lists the main symbols used in this paper.

### 3.4. Scheme Definition

A DFB-PPGSQ scheme consists of the following polynomial-time algorithms:

(2) Π=(KeyGen,Setup,UpdateToken,ApplyUpdate,TrapGen,Query,ReRand).

#### 3.4.1. KeyGen$(1^{\lambda })\to K$

Given the security parameter, the client generates master keys for encryption, pseudo-random functions, pseudo-random permutations, order or comparison modules, and epoch evolution.

#### 3.4.2. Setup$\left(K,G^{0}\right)\to \left({\mathrm{EDB}}_{0},I_{0},{\mathrm{st}}_{0}\right)$

The client encrypts the initial graph database, extracts branch multisets, builds the initial secure branch–tree index, and initializes the client state.

#### 3.4.3. UpdateToken(K,st,op)→Δ

For an insertion, deletion, or label update, the client generates an update token Δ. The token contains only encrypted feature handles and masked metadata needed by the cloud.

#### 3.4.4. ApplyUpdate$\left(\mathrm{EDB},I,U,D,\Delta \right)\to \left({\mathrm{EDB}}^{\prime },I^{\prime },U^{\prime },D^{\prime }\right)$

The cloud applies the update token. It inserts encrypted records into the update buffer, marks deleted records, or updates encrypted label-derived branch features.

#### 3.4.5. TrapGen$\left(K,\mathrm{st},Q,\tau \right)\to T_{Q}$

The client extracts the branch multiset of the query graph and generates a query token for the current epoch and active update buffers.

#### 3.4.6. Query$(\mathrm{EDB},I,U,D,T_{Q})\to R$

The cloud computes encrypted branch lower bounds, prunes candidate graphs using the branch–tree index, checks active-record states, and returns encrypted candidates.

#### 3.4.7. ReRand$\left(K,\mathrm{st},e\right)\to \Omega_{e}$

At an epoch boundary, the client generates a shuffled refresh package. The cloud replaces the old branch tree, active-record handles, and update buffers with the new package, but it does not receive an old-handle to new-handle correspondence table.

### 3.5. Privacy Goals

DFB-PPGSQ aims to achieve content privacy, query privacy, forward privacy, epochal backward privacy, and bounded re-randomization leakage.

#### 3.5.1. Content Privacy

The cloud should not learn plaintext graph structures, vertex labels, edge labels, query graphs, or update contents beyond the defined leakage functions.

#### 3.5.2. Query Privacy

The cloud should not learn the plaintext query graph. Depending on the selected security performance configuration, it may learn bounded query leakage such as result size and a protected traversal trace.

#### 3.5.3. Forward Privacy

Search tokens issued before an insertion or label update should not enable the cloud to identify whether the newly inserted or newly updated branch features match previous queries. In other words, future updates are protected from past searches.

#### 3.5.4. Backward Privacy

After a graph is deleted, future search tokens should not reveal information about that deleted graph beyond bounded deletion leakage. The strongest form hides the deleted graph from future results and search traces after compaction. A practical form allows temporary tombstone leakage within the current epoch and removes it at re-randomization.

#### 3.5.5. Re-Randomization Privacy

Across epochs, refreshed feature tokens, occurrence handles, tree-node handles, and active-record handles should not be linkable by the cloud except through explicitly leaked size buckets, update timing, and migration counts.

## 4. Preliminaries

### 4.1. Branch-Based Lower Bound of Edit Distance

A branch is a local graph feature centered at a vertex. It contains the center vertex label, the labels of incident edges, and the labels of neighboring vertices. The branch multiset of graph *G* is denoted by B(G).

For a branch *b* and a vertex label *ℓ*, the branch and vertex multiplicities of *G* are written as

(3) $$C_{B}\left(G,b\right)=|\left\{b^{\prime }\in B\left(G\right):b^{\prime }=b\right\}|,\;C_{V}\left(G,\ell \right)=\left|\left\{v\in V\left(G\right):L_{V}\left(v\right)=\ell \right\}\right|.$$

Let $B_{G,Q}=B\left(G\right)\cup B\left(Q\right)$ and $L_{G,Q}=L_{V}\left(G\right)\cup L_{V}\left(Q\right)$ be the union domains induced by a data graph and a query graph. The branch and vertex residuals are

(4) $$\Delta_{B}\left(G,Q\right)=\sum_{b\in B_{G,Q}}\left|C_{B}\left(G,b\right)-C_{B}\left(Q,b\right)\right|,$$

(5) $$\Delta_{V}\left(G,Q\right)=\sum_{\ell \in L_{G,Q}}\left|C_{V}\left(G,\ell \right)-C_{V}\left(Q,\ell \right)\right|.$$

Following the branch-based filtering principle, a computable lower-bound score is

(6) $$\mathrm{LBED}\left(G,Q\right)=\max\left\{\left\lceil \frac{\Delta_{B}\left(G,Q\right)}{2}\right\rceil ,\left\lceil \frac{\Delta_{V}\left(G,Q\right)}{2}\right\rceil \right\}.$$

If LBED(G,Q)>τ, then *G* cannot be a valid answer and can be pruned before exact refinement. This score is not used as a final graph edit distance estimator but as a safe filtering signal.

This filtering principle is central to PPGSQ-style systems. DFB-PPGSQ retains it. The new challenge is to maintain branch features under dynamic encrypted updates without exposing temporal correlations.

### 4.2. Structured Encryption

Structured encryption allows a client to outsource encrypted data structures while enabling controlled server-side operations [33]. A leakage function specifies what the server may learn during setup, update, and querying. Security is defined by simulation; whatever the real server observes should be simulatable from the allowed leakage.

DFB-PPGSQ treats the encrypted branch index as a structured encryption object. Branch identifiers are produced by pseudo-random functions, counters are masked or encrypted, tree nodes are re-randomized across epochs, and update tokens are generated from evolving keys.

The construction separates two token namespaces. In epoch *e*, the query-matchable feature token of branch *b* is

(7) $$\theta_{b}^{e}=F_{K_{F}^{e}}\left(\mathrm{canon}\left(b\right)\right),$$

where canon(b) is a canonical encoding of the branch. This token is deterministic only inside one epoch and is the object that query tokens can match. For storage, the client additionally derives a per-record occurrence handle

(8) $$\eta_{b,i,c}^{e}=F_{K_{O}^{e}}(\theta_{b}^{e}\;\|\;h_{i}^{e}\;\|\;c\;\|\;r_{i}),\;h_{i}^{e}=F_{K_{D}^{e}}\left({\mathrm{id}}_{i}\|\;e\right),$$

where $r_{i}$ is a graph-local nonce and *c* is an occurrence counter. The occurrence handle hides repeated occurrences and record membership; it is never used as the query key. This separation keeps feature matching well-defined while preventing the graph-local nonce from breaking query–token equality. The protected count stored at bucket $j=H_{K_{H}^{e}}\left(b\right)\;\mathrm{mod}\;m$ is

(9) $${\tilde{C}}_{G}^{e}\left[j\right]=C_{G}^{e}\left[j\right]+F_{K_{C}^{e}}\left({\mathrm{id}}_{G}\|\;j\right)\;\left(\mathrm{mod}\;p\right).$$

Only query-authorized masks can be canceled or compared. Thus, the cloud obtains enough information to run the filtering predicate but does not receive plaintext branches or stable cross-epoch identifiers.

### 4.3. Forward and Backward Privacy

Forward privacy prevents a server from linking future inserted data to previous search tokens. A common method is to use key evolution or one-time update labels. In DFB-PPGSQ, the update key for epoch *e* is derived through a one-way key chain

(10) $$K_{U}^{e,u}=F_{K_{U}^{e}}\left(\mathrm{upd}\|\;u\right),\;K_{U}^{e+1}=F_{K_{R}}\left(K_{U}^{e}\;\|\;e\right),$$

where *u* is a monotone update counter and $K_{R}$ is the re-randomization master key. The server cannot derive the label of a future update from a past search token.

Backward privacy controls leakage from deleted data. If a document, graph, or branch feature is deleted, later searches should not reveal it. Strong backward privacy can be expensive; therefore, DFB-PPGSQ offers an epochal design: within the current epoch, a deletion may leak a tombstone handle and active-state check, while stale handles are removed from the active searchable structure after re-randomization. Formally, for a deleted graph set $D_{e}$, query output is filtered by

(11) $$R_{e}\left(Q,\tau \right)=\left\{G_{i}:\mathrm{LBED}\left(G_{i},Q\right)\leq \tau \wedge h_{i}^{e}\notin D_{e}\right\}.$$

At re-randomization, only $G_{i}\notin D_{e}$ is migrated to $I_{e+1}$.

### 4.4. Periodic Re-Randomization

Periodic re-randomization refreshes encrypted identifiers and index nodes at epoch boundaries. This breaks long-term equality links and provides a natural moment for compaction at which deleted entries can be removed, update buffers can be merged, and counters can be regenerated. In the stronger refresh mode used by DFB-PPGSQ, the client prepares the next-epoch active summaries and uploads them in a fresh random order:

(12) $$\Omega_{e}=\pi_{e}\left(\left\{\left(h_{i}^{e+1},{\left\{\theta_{b}^{e+1},\eta_{b,i,c}^{e+1}\right\}}_{b\in B(G_{i})},{\tilde{C}}_{G_{i}}^{e+1}\right):h_{i}^{e}\in M_{e}\right\}\right),$$

where $\pi_{e}$ is a random permutation over active records. The cloud learns migration-size buckets, refreshed tree shape, and bucketed record sizes but does not receive a pairwise map from $h_{i}^{e}$ to $h_{i}^{e+1}$ or from $\theta_{b}^{e}$ to $\theta_{b}^{e+1}$ beyond the leakage function.

The epoch length controls a tradeoff in which a short epoch reduces temporal leakage but increases maintenance cost. A long epoch reduces maintenance cost but permits more accumulated leakage. Section 7 includes this tradeoff as an experimental variable.

## 5. The Proposed DFB-PPGSQ Scheme

### 5.1. Overview

DFB-PPGSQ follows the filtering-and-refinement philosophy. The client extracts branch features from data graphs and query graphs, the cloud uses an encrypted branch–tree index to filter candidates, and the client decrypts returned candidates; when exactness is required, the client refines the final graph edit distance locally.

At epoch *e*, the outsourced state is

(13) $${\mathrm{State}}_{e}=\left({\mathrm{EDB}}_{e},I_{e},U_{e},D_{e},{\mathrm{meta}}_{e}\right),$$

where ${\mathrm{EDB}}_{e}$ stores encrypted graph records, $I_{e}$ is the compact encrypted branch–tree index, $U_{e}$ is the recent update buffer, $D_{e}$ is the tombstone set, and ${\mathrm{meta}}_{e}$ contains non-secret size and epoch metadata allowed by the leakage function. The active encrypted graph handles satisfy the invariant

(14) $${\mathrm{Act}}_{e}=\left(\mathrm{Leaves}\left(I_{e}\right)\cup \mathrm{Handles}\left(U_{e}\right)\right)\setminus D_{e}.$$

The search procedure returns only handles in ${\mathrm{Act}}_{e}$.

The state invariant is maintained as follows: for every active logical graph ID, exactly one current encrypted record is addressable either through a compact index leaf or through the update buffer; every deletion handle in $D_{e}$ suppresses the corresponding active handle until refresh; every positive update label is generated with a non-reused counter; and every ReRand operation migrates only handles in ${\mathrm{Act}}_{e}$ into the next epoch and then clears $U_{e}$ and $D_{e}$. Thus, arbitrary insertion, deletion, label update, search, and refresh sequences preserve the relation among ${\mathrm{EDB}}_{e}$, $I_{e}$, $U_{e}$, and $D_{e}$ so long as the trusted client maintains the logical ID map and update counters.

**Figure 2:**
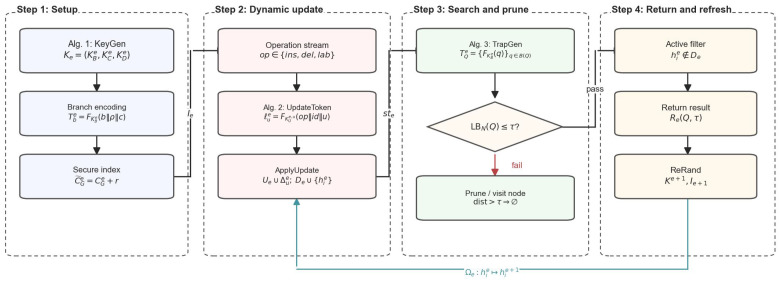
Algorithmic workflow of DFB-PPGSQ. The figure summarizes setup, dynamic update processing, encrypted graph similarity search and pruning, result return, and epoch refresh.

The construction evaluated in this paper is the lightweight configuration based on PRF tokens, masked counters, update buffers, tombstones, and shuffle refresh. Stronger tools such as homomorphic counters, ORAM traversal, volume padding, or oblivious compaction are compatible design directions but are not constructed or evaluated here, and are not claimed as part of the implemented scheme. As shown in Figure 2, the four dynamic mechanisms are as follows::

1. Epoch-local feature tokens and per-record occurrence handles for compact active data in $I_{e}$.
2. One-time update labels for recent insertions and label updates in $U_{e}$.
3. Epoch-specific deletion handles in $D_{e}$.
4. Re-randomization material $\Omega_{e}$ for epoch migration.

### 5.2. Branch Extraction, Token Generation, and Setup

For a graph $G=(V,E,L_{V},L_{E})$, the branch centered at v∈V is represented as

(15) $$b_{v}=\left(L_{V}\left(v\right),\left\{\;\left\{\left(L_{E}\left(v,u\right),L_{V}\left(u\right)\right):\left(v,u\right)\in E\right\}\;\right\}\right),$$

where {{·}} denotes a multiset. The graph branch multiset is

(16) $$B\left(G\right)=\{\;\left\{b_{v}:v\in V\right\}\;\}.$$

For each branch $b\in B(G_{i})$, the client generates an epoch-specific feature token and a per-record occurrence handle:

(17) $$\theta_{b}^{e}=F_{K_{F}^{e}}\left(\mathrm{canon}\left(b\right)\right),\;\eta_{b,i,c_{b}}^{e}=F_{K_{O}^{e}}(\theta_{b}^{e}\;\|\;h_{i}^{e}\;\|\;c_{b}\;\|\;r_{i}),$$

where *F* is a secure pseudo-random function, $K_{F}^{e}$ is the feature token key, $K_{O}^{e}$ is the occurrence-handle key, $h_{i}^{e}$ is the active graph handle, $r_{i}$ is a graph-local nonce, and $c_{b}$ is a local occurrence counter. Queries match $\theta_{b}^{e}$ values. The nonce appears only in $\eta_{b,i,c_{b}}^{e}$, so it randomizes stored occurrences without preventing query matching.

The client also prepares a protected branch count vector. A branch position is derived by

(18) $$j_{e}\left(b\right)=H_{K_{H}^{e}}\left(b\right)\;\mathrm{mod}\;m,\;x_{G}^{e}\left[j\right]=\sum_{b\in B(G)}1\left[j_{e}\left(b\right)=j\right],$$

and the protected count is

(19) $${\tilde{x}}_{G}^{e}\left[j\right]=x_{G}^{e}\left[j\right]+M_{K_{C}^{e}}\left({\mathrm{id}}_{G},j\right)\;\left(\mathrm{mod}\;p\right).$$

For a tree node ν containing graph handles $S_{\nu }$, the aggregate plaintext count vector is

(20) $$x_{\nu }^{e}\left[j\right]=\max_{G_{i}\in S_{\nu }}x_{G_{i}}^{e}\left[j\right],$$

and the stored value is masked in the same way. The maximum aggregate makes the node a safe filter: if a query cannot match the aggregate, it cannot match any child graph under the same lower-bound rule.

Algorithm 1 shows that setup is not merely an encryption step; it also initializes the epoch keys, graph nonces, masked branch counts, and protected node aggregates required for later pruning. The server sees protected handles and vector lengths, while the client keeps the semantic map from plaintext branches to epoch-local feature tokens.
**Algorithm 1** Setup and Protected Branch–Tree Construction**Input:** master key *K*, initial graph database $G^{0}=\left\{G_{1},\ldots ,G_{N}\right\}$, vector length *m*, modulus *p*
**Output:** encrypted database ${\mathrm{EDB}}_{0}$, branch-tree index $I_{0}$, client state ${\mathrm{st}}_{0}$
  1: Derive epoch keys $\left(K_{F}^{0},K_{O}^{0},K_{H}^{0},K_{C}^{0},K_{U}^{0},K_{D}^{0}\right)\leftarrow F_{K}\left(\mathrm{setup}\|\;0\right)$  2: Initialize ${\mathrm{EDB}}_{0}\leftarrow ⌀$, $S_{0}\leftarrow ⌀$, $U_{0}\leftarrow ⌀$, $D_{0}\leftarrow ⌀$  3: **for** each graph $G_{i}\in G^{0}$ **do**  4:       sample graph nonce $r_{i}\leftarrow {\left\{0,1\right\}}^{\lambda }$ and handle $h_{i}^{0}\leftarrow F_{K_{D}^{0}}\left({\mathrm{id}}_{i}\|\;0\right)$  5:       compute branch multiset $B(G_{i})$ and count vector $x_{G_{i}}^{0}$  6:       **for** each occurrence $\left(b,c_{b}\right)\in B\left(G_{i}\right)$ **do**  7:             generate feature token $\theta_{b}^{0}\leftarrow F_{K_{F}^{0}}\left(\mathrm{canon}\left(b\right)\right)$  8:             generate occurrence handle $\eta_{b,i,c_{b}}^{0}\leftarrow F_{K_{O}^{0}}(\theta_{b}^{0}\;\|\;h_{i}^{0}\;\|\;c_{b}\;\|\;r_{i})$  9:       **end for**10:       mask counts ${\tilde{x}}_{G_{i}}^{0}\left[j\right]\leftarrow x_{G_{i}}^{0}\left[j\right]+M_{K_{C}^{0}}\left({\mathrm{id}}_{i},j\right)\;\mathrm{mod}\;p$11:       append ${\mathrm{Enc}}_{K}\left(G_{i}\right)$ to ${\mathrm{EDB}}_{0}$ and $\left(h_{i}^{0},\left\{\left(\theta_{b}^{0},\eta_{b,i,c_{b}}^{0}\right)\right\},{\tilde{x}}_{G_{i}}^{0}\right)$ to $S_{0}$12: **end for**13: build $I_{0}\leftarrow \mathrm{BuildTree}\left(S_{0}\right)$ with node aggregates ${\tilde{x}}_{\nu }^{0}$14: set ${\mathrm{st}}_{0}\leftarrow \left(K,0,{\left\{r_{i}\right\}}_{i=1}^{N},\mathrm{ctr}=0\right)$15: **return** $\left({\mathrm{EDB}}_{0},I_{0},{\mathrm{st}}_{0}\right)$


### 5.3. Dynamic Branch–Tree Index

The branch–tree index groups graphs by encrypted branch summary ranges. Each internal node stores a protected aggregate summary of its children, and each leaf stores encrypted graph handles and compact branch count summaries. BuildTree is a client-side protected tree construction routine. Given active summaries, the client first applies a keyed pseudo-random ordering or the refresh permutation, packs leaves with a fixed parity parameter *a*, computes each internal node aggregate as the coordinate-wise maximum of its child plaintext count vectors before masking, and uploads only protected node summaries, child pointers, and size metadata allowed by the leakage function. The cloud stores and traverses this protected tree but does not need plaintext labels to construct node aggregates.

For a query branch count vector $x_{Q}^{e}$ and a node aggregate $x_{\nu }^{e}$, define the residual branch deficit and the residual vertex count deficit as

(21) $$\Delta_{B}\left(Q,\nu \right)=\sum_{j=1}^{m}\max\left\{0,x_{Q}^{e}\left[j\right]-x_{\nu }^{e}\left[j\right]\right\},\;\Delta_{V}\left(Q,\nu \right)=\max\{0,|V_{Q}\left|-V_{\nu }^{\max}\right\}.$$

The node lower bound follows the same max-based form as the graph-level lower bound:

(22) $${\mathrm{LBED}}_{e}\left(Q,\nu \right)=\max\left\{\left\lceil \frac{\Delta_{B}\left(Q,\nu \right)}{2}\right\rceil ,\left\lceil \frac{\Delta_{V}\left(Q,\nu \right)}{2}\right\rceil \right\}.$$

If encrypted vertex label aggregates are enabled, $\Delta_{V}\left(Q,\nu \right)$ can instead use the same positive-deficit sum over vertex label counters; the prototype uses the conservative vertex-count version. A node is safely pruned when

(23) $${\mathrm{LBED}}_{e}\left(Q,\nu \right)>\tau .$$

For any child graph $G_{i}$ under ν, the aggregate construction gives $x_{G_{i}}^{e}\left[j\right]\leq x_{\nu }^{e}\left[j\right]$, so

(24) $${\mathrm{LBED}}_{e}\left(Q,\nu \right)\leq {\mathrm{LBED}}_{e}\left(Q,G_{i}\right).$$

Thus, the pruning rule cannot discard a graph for which the exact branch lower bound is at most τ.

### 5.4. Forward-Private Insertion

When inserting a graph $G_{\mathrm{new}}$, the client extracts $B(G_{\mathrm{new}})$, derives feature tokens $\theta_{b}^{e}$, generates a fresh graph nonce $r_{\mathrm{new}}$, and creates one-time update labels with a monotonically increasing counter *u*:

(25) $$U_{b,u}^{e}=F_{K_{U}^{e}}\left(\theta_{b}^{e}\;\|\;u\right).$$

The counter is never re-used. The update record is

(26) $$\Delta_{\mathrm{ins}}=\left(\mathrm{ins},h_{\mathrm{new}}^{e},{\mathrm{Enc}}_{K}\left(G_{\mathrm{new}}\right),{\left\{U_{b,u}^{e}\right\}}_{b\in B(G_{\mathrm{new}})},{\tilde{x}}_{\mathrm{new}}^{e}\right).$$

The cloud appends this record to $U_{e}$. Since $U_{b,u}^{e}$ is derived from an update key and a future counter, an old query token cannot be used to test whether a future inserted graph contains a branch matching that old query. A later query authorizes only the current update counter interval needed to test buffered records.

### 5.5. Backward-Private Deletion

When deleting graph $G_{i}$, the client issues an epochal deletion handle

(27) $$h_{i}^{e}=F_{K_{D}^{e}}\left({\mathrm{id}}_{i}\|\;e\right).$$

The deletion token is

(28) $$\Delta_{\mathrm{del}}=\left(\mathrm{del},h_{i}^{e}\right),\;D_{e}\leftarrow D_{e}\cup \left\{h_{i}^{e}\right\}.$$

During the same epoch, deletion leaks only the tombstone handle and the fact that a candidate may be suppressed by $D_{e}$. At re-randomization, deleted records are not migrated into $I_{e+1}$; after that point, they no longer participate in future search traces.

### 5.6. Label Update

A label update modifies vertex labels or edge labels in a graph. It changes only those branches for which the center or one-hop neighborhood intersects the edited element. If δ is an edit operation on $G_{i}$, define the affected branch set as

(29) $$\mathrm{Aff}\left(G_{i},\delta \right)=B\left(G_{i}\right)▵B\left(G_{i}⊕\delta \right),$$

where ▵ is multiset symmetric difference. The update decomposes into removals of old branches and insertions of new branches:

(30) $${\mathrm{Aff}}^{-}=\mathrm{Aff}\cap B\left(G_{i}\right),\;{\mathrm{Aff}}^{+}=\mathrm{Aff}\cap B\left(G_{i}⊕\delta \right).$$

For each $b\in {\mathrm{Aff}}^{+}$, the client creates a fresh one-time update label $U_{b,u}^{e}$, while for each $b\in {\mathrm{Aff}}^{-}$ the client creates a removal tag tied to the active handle. The protected delta vector is

(31) $$\Delta {\tilde{x}}_{i}^{e}\left[j\right]=\left(x_{G_{i}⊕\delta }^{e}\left[j\right]-x_{G_{i}}^{e}\left[j\right]\right)+M_{K_{C}^{e}}\left({\mathrm{id}}_{i},j,u\right)\;\left(\mathrm{mod}\;p\right).$$

Algorithm 2 is the unified dynamic update interface. Insertions and positive label deltas use fresh update labels, while deletions use epochal tombstones; label changes are represented as local branch deltas. This keeps ordinary updates small and postpones tree rebuilding to epoch maintenance.
**Algorithm 2** Dynamic Graph Update Processing**Input:** operation op, epoch *e*, client state ${\mathrm{st}}_{e}$, outsourced state $\left({\mathrm{EDB}}_{e},I_{e},U_{e},D_{e}\right)$ **Output:** updated outsourced state and client state
  1: **if** op=Insert(G) **then**  2:       sample graph nonce $r_{G}$ and compute B(G), ${\tilde{x}}_{G}^{e}$, and active handle $h_{G}^{e}$  3:       generate feature tokens ${\left\{\theta_{b}^{e}\right\}}_{b\in B(G)}$ and one-time update labels ${\left\{U_{b,u}^{e}\right\}}_{b\in B(G)}$ with fresh counter values  4:       $\Delta \leftarrow (\mathrm{ins},h_{G}^{e},{\mathrm{Enc}}_{K}\left(G\right),\left\{U_{b,u}^{e}\right\},{\tilde{x}}_{G}^{e})$  5:       append Δ to $U_{e}$ and ${\mathrm{Enc}}_{K}\left(G\right)$ to ${\mathrm{EDB}}_{e}$  6: **else if** op=Delete(id) **then**  7:       compute $h_{\mathrm{id}}^{e}\leftarrow F_{K_{D}^{e}}\left(\mathrm{id}\|e\right)$  8:       append $h_{\mathrm{id}}^{e}$ to $D_{e}$  9: **else if** op=UpdateLabel(id,δ) **then**10:       compute ${\mathrm{Aff}}^{-}$ and ${\mathrm{Aff}}^{+}$ for $G_{\mathrm{id}}⊕\delta$11:       generate removal tags for ${\mathrm{Aff}}^{-}$ and one-time update labels for ${\mathrm{Aff}}^{+}$12:       compute masked delta vector $\Delta {\tilde{x}}_{\mathrm{id}}^{e}$13:       append $\left(\mathrm{upd},h_{\mathrm{id}}^{e},{\mathrm{Aff}}^{-},{\mathrm{Aff}}^{+},\Delta {\tilde{x}}_{\mathrm{id}}^{e}\right)$ to $U_{e}$14: **end if**15: update local counters in ${\mathrm{st}}_{e}$16: **return** $\left({\mathrm{EDB}}_{e},I_{e},U_{e},D_{e},{\mathrm{st}}_{e}\right)$


### 5.7. Query Processing

Given query graph *Q* and threshold τ, the client extracts B(Q) and generates a query token

(32) $$T_{Q}^{e}=\left({\left\{\theta_{q}^{e}\right\}}_{q\in B(Q)},{\left\{\omega_{q,u}^{e}\right\}}_{q\in B\left(Q\right),u\in U_{e}},{\tilde{x}}_{Q}^{e},\tau ,{\mathrm{auth}}_{e}\right),$$

where $\omega_{q,u}^{e}=F_{K_{U}^{e}}\left(\theta_{q}^{e}\;\|\;u\right)$ authorizes comparisons with buffered update labels for the current update counter interval $U_{e}$. Thus, $\theta_{q}^{e}$ searches the compact branch tree $I_{e}$, while $\omega_{q,u}^{e}$ searches one-time labels in $U_{e}$. The cloud computes two candidate sets:

(33) $${\mathrm{Cand}}_{I}=\left\{h_{i}^{e}\in I_{e}:{\mathrm{LBED}}_{e}\left(Q,G_{i}\right)\leq \tau \right\},$$

(34) $${\mathrm{Cand}}_{U}=\left\{h_{i}^{e}\in U_{e}:{\mathrm{LBED}}_{e}\left(Q,G_{i}\right)\leq \tau \right\}.$$

The active candidate set is

(35) $${\mathrm{Cand}}_{e}\left(Q,\tau \right)=\left({\mathrm{Cand}}_{I}\cup {\mathrm{Cand}}_{U}\right)\setminus D_{e}.$$

The cloud never sees *Q* or plaintext graph labels. It observes the declared traversal pattern, update buffer checks, and result size. If the application requires less traversal leakage, the same query interface can be combined with padded traversal or oblivious access at additional cost.

The node-level primitive operations of nodes in Algorithm 3 are as shown in Algorithm 4. It no longer introduces a separate 0.5t vertex-token term; instead, it computes the same max-based residual form used in Section 4. The early termination rule is safe because $\Delta_{B}$ is a sum of non-negative coordinate deficits; once $\lceil \Delta_{B}/2\rceil >\tau$, later coordinates cannot reduce the lower bound below the pruning threshold. This makes the relationship between the pruning algorithm and $\mathrm{LBED}\left(G,Q\right)=\max\{\left\lceil \Delta_{B}/2\right\rceil ,\left\lceil \Delta_{V}/2\right\rceil \}$ explicit.
**Algorithm 3** Encrypted Graph Similarity Search**Input:** query graph *Q*, threshold τ, epoch *e*, state $\left(I_{e},U_{e},D_{e}\right)$ **Output:** encrypted candidate set $R_{e}\left(Q,\tau \right)$
  1: Client computes B(Q), ${\tilde{x}}_{Q}^{e}$, and query token $T_{Q}^{e}$  2: Server initializes ${\mathrm{Cand}}_{I}\leftarrow ⌀$ and queue $Q\leftarrow \{\mathrm{root}\left(I_{e}\right)\}$  3: **while** Q≠⌀ **do**  4:       pop node ν from Q  5:       compute ${\mathrm{dist}}_{\nu }\leftarrow {\mathrm{LBED}}_{e}\left(Q,\nu \right)$ using authorized protected summaries  6:       **if** ${\mathrm{dist}}_{\nu }\leq \tau$ and ν is internal **then**  7:             push all children of ν into Q  8:       **else if** ${\mathrm{dist}}_{\nu }\leq \tau$ and ν is a leaf **then**  9:             test graph-level summaries in ν and append passing handles to ${\mathrm{Cand}}_{I}$10:       **end if**11: **end while**12: compute ${\mathrm{Cand}}_{U}$ by testing buffered insertions and label-update deltas in $U_{e}$13: filter tombstones: ${\mathrm{Cand}}_{e}\leftarrow \left({\mathrm{Cand}}_{I}\cup {\mathrm{Cand}}_{U}\right)\setminus D_{e}$14: return encrypted graph records $R_{e}\left(Q,\tau \right)\leftarrow \left\{{\mathrm{EDB}}_{e}\left[h\right]:h\in {\mathrm{Cand}}_{e}\right\}$


**Algorithm 4** Node Lower-Bound Test**Input:** query count vector $x_{Q}^{e}$, node aggregate $x_{\nu }^{e}$, query vertex count $|V_{Q}|$, node maximum vertex count $V_{\nu }^{\max}$, threshold τ **Output:** lower-bound score ${\mathrm{dist}}_{\nu }$ for node pruning
  1: Initialize $\Delta_{B}\leftarrow 0$  2: **for** j=1 to *m* **do**  3:       $\delta_{j}\leftarrow \max\left\{0,x_{Q}^{e}\left[j\right]-x_{\nu }^{e}\left[j\right]\right\}$  4:       $\Delta_{B}\leftarrow \Delta_{B}+\delta_{j}$  5:       **if** $\left\lceil \Delta_{B}/2\right\rceil >\tau$ **then**  6:             **return** $\left\lceil \Delta_{B}/2\right\rceil$  7:       **end if**  8: **end for**  9: $\Delta_{V}\leftarrow \max\{0,|V_{Q}\left|-V_{\nu }^{\max}\right\}$10: ${\mathrm{dist}}_{\nu }\leftarrow \max\left\{\left\lceil \Delta_{B}/2\right\rceil ,\left\lceil \Delta_{V}/2\right\rceil \right\}$11: **return** ${\mathrm{dist}}_{\nu }$


### 5.8. Periodic Re-Randomization

At the end of epoch *e*, the client starts ReRand. It derives new epoch keys

(36) $$\left(K_{F}^{e+1},K_{O}^{e+1},K_{H}^{e+1},K_{C}^{e+1},K_{U}^{e+1},K_{D}^{e+1}\right)=F_{K}\left(\mathrm{epoch}\|\;e+1\right).$$

Let the active migration set be

(37) $$M_{e}=\left\{h_{i}^{e}:h_{i}^{e}\in \mathrm{Leaves}\left(I_{e}\right)\cup \mathrm{Handles}\left(U_{e}\right),\;h_{i}^{e}\notin D_{e}\right\}.$$

For every active graph, as shown in Algorithm 5, the client computes new feature tokens, occurrence handles, active handles, and masked counters under epoch e+1 keys. The refresh package is

(38) $$\Omega_{e}=\pi_{e}\left(\{\left(h_{i}^{e+1},{\left\{\left(\theta_{b}^{e+1},\eta_{b,i,c_{b}}^{e+1}\right)\right\}}_{b\in B(G_{i})},{\tilde{x}}_{i}^{e+1}\right):h_{i}^{e}\in M_{e}\}\right),$$

where $\pi_{e}$ is sampled by the client. The cloud installs this package as a replacement state, meaning that it observes the number of active records and bucketed sizes but not a pairwise old-to-new handle map.
**Algorithm 5** Epoch Re-Randomization and State Refresh**Input:** epoch *e*, index $I_{e}$, update buffer $U_{e}$, tombstones $D_{e}$, client state ${\mathrm{st}}_{e}$
**Output:**
refreshed index $I_{e+1}$ and cleared buffers
  1: Client derives $\left(K_{F}^{e+1},K_{O}^{e+1},K_{H}^{e+1},K_{C}^{e+1},K_{U}^{e+1},K_{D}^{e+1}\right)$  2: Client determines active logical ids represented by $M_{e}$ and excludes $D_{e}$  3: Initialize local refresh list $S_{e+1}\leftarrow ⌀$  4: **for** each active graph id ${\mathrm{id}}_{i}$ in random order $\pi_{e}$ **do**  5:       compute $h_{i}^{e+1}\leftarrow F_{K_{D}^{e+1}}\left({\mathrm{id}}_{i}\|\;e+1\right)$  6:       refresh feature tokens $\theta_{b}^{e+1}\leftarrow F_{K_{F}^{e+1}}\left(\mathrm{canon}\left(b\right)\right)$  7:       refresh occurrence handles $\eta_{b,i,c_{b}}^{e+1}\leftarrow F_{K_{O}^{e+1}}(\theta_{b}^{e+1}\|h_{i}^{e+1}\|c_{b}\left\|r_{i}\right)$  8:       recompute or remask ${\tilde{x}}_{i}^{e+1}$  9:       append $\left(h_{i}^{e+1},\left\{\left(\theta_{b}^{e+1},\eta_{b,i,c_{b}}^{e+1}\right)\right\},{\tilde{x}}_{i}^{e+1}\right)$ to $S_{e+1}$10: **end for**11: Client computes protected node aggregates and sends a shuffled tree package $\Omega_{e}=\mathrm{BuildTree}\left(S_{e+1}\right)$12: Server installs $I_{e+1}\leftarrow \Omega_{e}$ without receiving an old-to-new handle map13: set $U_{e+1}\leftarrow ⌀$, $D_{e+1}\leftarrow ⌀$, and securely discard stale epoch material14: update client state ${\mathrm{st}}_{e+1}$15: **return** $\left(I_{e+1},U_{e+1},D_{e+1},{\mathrm{st}}_{e+1}\right)$


Re-randomization can be full or incremental. Full re-randomization rebuilds the whole active index from a shuffled client refresh package. Incremental re-randomization uploads a fixed budget of refreshed records per time window, with a fresh permutation inside each batch. In both cases, deleted records are excluded from $M_{e}$, which is the mechanism that gives the construction its epochal backward-privacy effect. The incremental mode leaks batch sizes and batch timing; the full mode leaks the epoch refresh size but avoids pairwise migration linkage.

## 6. Security Analysis

### 6.1. Threat Model and Leakage Profile

We model the cloud as an honest-but-curious probabilistic polynomial-time adversary. It follows Setup, ApplyUpdate, Query, and ReRand but tries to infer graph contents, query branches, update contents, and temporal relations among operations. The client is trusted and keeps secret the master key *K*, epoch keys, graph nonces, logical ID map, and local counters. The encrypted graph database is protected by an IND-CPA secure symmetric encryption scheme, and all token-generation functions are modeled as pseudo-random functions.

The security goal is leakage-based structured encryption security. Let

(39) $$O=(\mathrm{setup},{\mathrm{op}}_{1},\ldots ,{\mathrm{op}}_{T})$$

be an adaptive operation sequence selected by the adversary, where each ${\mathrm{op}}_{t}$ is an insertion, deletion, label update, query, or epoch refresh. The real server view is

(40) $${\mathrm{View}}_{A}^{\Pi }\left(\lambda ,O\right)=\left({\mathrm{EDB}}_{e},I_{e},U_{e},D_{e},{\left\{\Delta_{t}\right\}}_{t\leq T},{\left\{T_{Q_{t}}^{e_{t}}\right\}}_{t\leq T},{\left\{{\mathrm{path}}_{e_{t}}\left(Q_{t}\right),R_{e_{t}}\left(Q_{t},\tau_{t}\right)\right\}}_{t\leq T}\right).$$

The simulator is allowed to see only a leakage function

(41) $$L=\left(L_{\mathrm{setup}},L_{\mathrm{upd}},L_{\mathrm{qry}},L_{\mathrm{rr}}\right).$$

**Definition 1** 
(Leakage functions)**.** *For an initial database $G^{0}$,update ${\mathrm{op}}_{t}$, query $\left(Q_{t},\tau_{t}\right)$, and epoch refresh from e to e+1, DFB-PPGSQ exposes the following leakage:*

(42) $$L_{\mathrm{setup}}\left(G^{0}\right)=\left(N,\{|G_{i}{\left|\right\}}_{i=1}^{N},\{|B\left(G_{i}\right){\left|\right\}}_{i=1}^{N},m,p,\mathrm{shape}\left(I_{0}\right),\{|S_{\nu }{\left|\right\}}_{\nu \in I_{0}}\right),$$

(43) $$L_{\mathrm{upd}}\left({\mathrm{op}}_{t},e\right)=\left(t,e,\mathrm{type}\left({\mathrm{op}}_{t}\right),\mathrm{bucket}(|\Delta_{t}|),\mathrm{bucket}(|\mathrm{Aff}\left({\mathrm{op}}_{t}\right)\left|\right),1\left[{\mathrm{op}}_{t}=\mathrm{del}\right]\right),$$

(44) $$L_{\mathrm{qry}}\left(Q_{t},\tau_{t},e\right)=\left(t,e,\tau_{t},{\mathrm{path}}_{e}\left(Q_{t}\right),{\mathrm{updscan}}_{e}\left(Q_{t}\right),|{\mathrm{Cand}}_{e}\left(Q_{t},\tau_{t}\right)\left|,\right|R_{e}\left(Q_{t},\tau_{t}\right)|\right),$$

*and*

(45) $$L_{\mathrm{rr}}\left(e\right)=\left(e,|M_{e}\left|,\right|D_{e}\left|,\right|U_{e}|,\mathrm{shape}\left(I_{e+1}\right),\mathrm{bucketmultiset}(\{|\mathrm{EDB}\left[h\right]|\;:h\in M_{e}\}),\mathrm{bucket}\left(T_{\mathrm{rr}}\right)\right).$$

*Here, N is the number of outsourced graphs, m and p are public counter parameters, $S_{\nu }$ is the handle set under node ν, Aff is the affected branch-delta set for a label update, ${\mathrm{path}}_{e}\left(Q_{t}\right)$ is the tree traversal trace, and ${\mathrm{updscan}}_{e}\left(Q_{t}\right)$ is the set of update buffer records tested by the query token.*

The leakage definition is intentionally explicit. DFB-PPGSQ does not claim oblivious access, result-size hiding, or fully hidden update timing; instead, it prevents the cloud from learning plaintext graph labels, plaintext branch equality across protected epochs, and token-level relations beyond the declared size, timing, traversal, and migration metadata. This leakage-based theorem proves that the real view reveals no more than L under the stated cryptographic assumptions; it does not prove that every observable in L is harmless against arbitrary auxiliary information, statistical, or machine learning leakage abuse attacks, which must be evaluated separately for a concrete deployment profile.

### 6.2. Real–Ideal Security Definition

**Definition 2** 
(Adaptive leakage security)**.** *Let ${\mathrm{Real}}_{A}^{\Pi }\left(\lambda \right)$ be the experiment in which adversary A adaptively interacts with the real DFB-PPGSQ protocol and receives the real server view. Let ${\mathrm{Ideal}}_{A,S}^{L}\left(\lambda \right)$ be the experiment in which A interacts with a simulator S that receives only L(O) for the same operation sequence. DFB-PPGSQ is adaptively secure with respect to L if, for every PPT adversary A, there exists a PPT simulator S such that*

(46) $${\mathrm{Adv}}_{\Pi ,L}^{\mathrm{se}}\left(A,S,\lambda \right)=\left|\mathrm{Pr}\left[A\left({\mathrm{View}}_{\mathrm{real}}\right)=1\right]-\mathrm{Pr}\left[A\left({\mathrm{View}}_{\mathrm{ideal}}\right)=1\right]\right|\leq \mathrm{negl}\left(\lambda \right).$$

The simulator uses $L_{\mathrm{setup}}$ to sample ciphertext strings, protected handles, vector lengths, and a tree with the leaked shape. It uses $L_{\mathrm{upd}}$ to sample update records with the correct operation class and size bucket. It uses $L_{\mathrm{qry}}$ to reproduce the same pattern of visited nodes, update buffer scan size, candidate size, and returned ciphertext count. It uses $L_{\mathrm{rr}}$ to sample shuffled epoch refresh messages and the new tree shape without using a pairwise old-to-new handle or branch map.

### 6.3. Token and Ciphertext Indistinguishability

**Lemma 1** 
(Pseudo-random token simulation)**.** *Let F be a secure pseudo-random function. For any polynomial number of inputs*

(47) $$x_{r}\in \{\mathrm{canon}\left(b\right),\;\theta_{b}^{e}\;\|\;h_{i}^{e}\;\|\;c_{b}\;\|\;r_{i},\;\theta_{b}^{e}\;\|\;u,\;{\mathrm{id}}_{i}\;\|\;e,\;\mathrm{epoch}\|\;e\},$$

*the token family ${\left\{F_{K}\left(x_{r}\right)\right\}}_{r}$ is computationally indistinguishable from random strings of the same length, preserving only equality that is intentionally leaked by same-epoch feature tokens or authorized update probes. More precisely, for every PPT distinguisher D,*

(48) $$\left|\mathrm{Pr}\left[D\left({\left\{F_{K}\left(x_{r}\right)\right\}}_{r}\right)=1\right]-\mathrm{Pr}\left[D\left({\left\{U_{r}\right\}}_{r}\right)=1\right]\right|\leq {\mathrm{Adv}}_{F}^{\mathrm{prf}}\left(B,\lambda \right),$$

*where ${\left\{U_{r}\right\}}_{r}$ are independent uniform strings and B is a standard PRF adversary constructed from D.*

**Proof.**  The proof is the standard PRF hybrid. Replace the oracle used to answer each token request with a truly random function. Feature token equality for the same branch inside one epoch is intentionally retained because it is needed for matching; occurrence handles, update labels, deletion handles, and epoch identifiers use non-reused namespaces. If D distinguishes real tokens from random labels under these retained equality constraints, then D directly yields a PRF distinguisher B with the same distinguishing gap. Thus, the difference is bounded by ${\mathrm{Adv}}_{F}^{\mathrm{prf}}\left(B,\lambda \right)$.    □

**Lemma 2** 
(Encrypted record simulation)**.** *If the graph encryption scheme is IND-CPA secure, then encrypted graph records in ${\mathrm{EDB}}_{e}$ can be simulated from record-size leakage. For any PPT adversary A,*

(49) $$\left|\mathrm{Pr}\left[A\left({\left\{{\mathrm{Enc}}_{K}\left(G_{i}\right)\right\}}_{i}\right)=1\right]-\mathrm{Pr}\left[A\left({\left\{{\mathrm{Enc}}_{K}\left(0^{|G_{i}|}\right)\right\}}_{i}\right)=1\right]\right|\leq {\mathrm{Adv}}_{\mathrm{Enc}}^{\mathrm{ind}-\mathrm{cpa}}\left(B,\lambda \right).$$

**Proof.**  The simulator encrypts zero strings of the same size buckets as the real graph encodings. Any adversary that distinguishes these simulated ciphertexts from real graph ciphertexts breaks IND-CPA security through the usual many-message hybrid.    □

### 6.4. Main Security Theorem

**Theorem 1** 
(Structured encryption security of DFB-PPGSQ)**.** *Assume that F is a secure pseudo-random function, $M_{K_{C}^{e}}$ is a pseudo-random mask family over $Z_{p}$, graph encryption is IND-CPA secure, and nonces/counters are non-reused within their namespaces. Then, DFB-PPGSQ is adaptively secure with respect to the leakage function L in Definition 1:*

(50) $${\mathrm{Adv}}_{\Pi ,L}^{\mathrm{se}}\left(A,S,\lambda \right)\leq {\mathrm{Adv}}_{\mathrm{Enc}}^{\mathrm{ind}-\mathrm{cpa}}+q_{F}{\mathrm{Adv}}_{F}^{\mathrm{prf}}+q_{M}{\mathrm{Adv}}_{M}^{\mathrm{prf}}+\mathrm{negl}\left(\lambda \right),$$

*where $q_{F}$ and $q_{M}$ are polynomially bounded numbers of token and mask queries.*

**Proof.**  We use a sequence of hybrids. In $H_{0}$, the adversary receives the real server view. In $H_{1}$, every encrypted graph record ${\mathrm{Enc}}_{K}\left(G_{i}\right)$ is replaced by an encryption of a zero string with the same leaked size. By Lemma 2, $H_{0}$ and $H_{1}$ are indistinguishable under IND-CPA security.

In $H_{2}$, all feature tokens, occurrence handles, update labels, deletion handles, query labels, and epoch keys are replaced by random strings that preserve equality only when equality is allowed by L in the same epoch or by a query-authorized update probe. Lemma 1 bounds the distinguishing advantage between $H_{1}$ and $H_{2}$ by the PRF advantage.

In $H_{3}$, protected count values

(51) $${\tilde{x}}_{G}^{e}\left[j\right]=x_{G}^{e}\left[j\right]+M_{K_{C}^{e}}\left({\mathrm{id}}_{G},j\right)\;\left(\mathrm{mod}\;p\right)$$

and node aggregates are replaced by random elements of $Z_{p}$ that are consistent with the leaked vector length, modulus, and comparison outcomes. Since $M_{K_{C}^{e}}$ is pseudo-random, the adversary sees only masked values and the declared pruning outcomes. Thus, $H_{2}$ and $H_{3}$ are indistinguishable up to ${\mathrm{Adv}}_{M}^{\mathrm{prf}}$.

In $H_{4}$, the simulator generates the entire server view using L. It samples ciphertexts with leaked sizes, random handles and tokens with leaked equality patterns, a tree with shape $\mathrm{shape}(I_{e})$, query traversal paths ${\mathrm{path}}_{e}\left(Q\right)$, update scans ${\mathrm{updscan}}_{e}\left(Q\right)$, result counts, and shuffled epoch refresh sizes. By construction, $H_{3}$ and $H_{4}$ have the same distribution; therefore, the real and ideal experiments are computationally indistinguishable except for the stated cryptographic advantages.    □

### 6.5. Forward Privacy

**Theorem 2** 
(Forward privacy)**.** *Let t be a time before an insertion or positive label-update delta at time $t^{\prime }>t$. Under the PRF assumption and non-reused update counters, any token view observed before $t^{\prime }$ is independent of the plaintext branch set inserted at $t^{\prime }$ except for $L_{\mathrm{upd}}$. Formally, for every branch predicate P and PPT adversary A,*

(52) $$\left|\mathrm{Pr}\left[A\left({\mathrm{View}}_{<t^{\prime }},\Delta_{t^{\prime }}\right)=P\left(B_{\mathrm{new}}\right)\right]-\mathrm{Pr}\left[A\left({\mathrm{View}}_{<t^{\prime }},{\hat{\Delta }}_{t^{\prime }}\right)=P\left(B_{\mathrm{new}}\right)\right]\right|\leq {\mathrm{Adv}}_{F}^{\mathrm{prf}}+\mathrm{negl}\left(\lambda \right),$$

*where ${\hat{\Delta }}_{t^{\prime }}$ is a simulated update record with the same leakage but random update labels.*

**Proof.**  For each inserted branch $b\in B_{\mathrm{new}}$, the server receives

(53) $$U_{b,u}^{e}=F_{K_{U}^{e}}\left(\theta_{b}^{e}\;\|\;u\right),$$

where *u* has not appeared before. Tokens issued before $t^{\prime }$ did not contain the future counter probe $\omega_{b,u}^{e}=F_{K_{U}^{e}}\left(\theta_{b}^{e}\;\|\;u\right)$. Hence, there is no efficiently testable relation between an old search token and $U_{b,u}^{e}$ unless a future query intentionally authorizes that update counter interval. Replacing $U_{b,u}^{e}$ with a random string changes the adversary’s view only by the PRF advantage. The remaining visible information is update time, type, epoch, and size bucket, which is exactly $L_{\mathrm{upd}}$.    □

### 6.6. Backward Privacy

**Theorem 3** 
(Epochal backward privacy)**.** *Let the graph handle $h_{i}^{e}$ be deleted during epoch e, and let the next refresh produce epoch e+1. If deleted handles are excluded from $M_{e}$, then future search views in epochs $e^{\prime }>e$ are independent of $G_{i}$ beyond deletion and migration leakage:*

(54) $$h_{i}^{e}\in D_{e}\;⟹\;h_{i}^{e+1}\notin \mathrm{Leaves}\left(I_{e+1}\right)\cup \mathrm{Handles}\left(U_{e+1}\right),$$

*and for every future query (Q,τ),*

(55) $$\mathrm{Pr}[h_{i}^{e+1}\in {\mathrm{Cand}}_{e+1}\left(Q,\tau \right)]=0.$$

**Proof.**  During epoch *e*, the deletion operation adds

(56) $$\Delta_{\mathrm{del}}=\left(\mathrm{del},h_{i}^{e}\right),\;D_{e}\leftarrow D_{e}\cup \left\{h_{i}^{e}\right\}.$$

Same-epoch queries may reveal that a candidate is suppressed by a tombstone; this is included in the deletion leakage. At ReRand, the migration set is

(57) $$M_{e}=\left(\mathrm{Leaves}\left(I_{e}\right)\cup \mathrm{Handles}\left(U_{e}\right)\right)\setminus D_{e}.$$

Thus, $h_{i}^{e}\notin M_{e}$, and no new active handle $h_{i}^{e+1}$, occurrence handle $\eta_{b,i,c_{b}}^{e+1}$, or protected count vector ${\tilde{x}}_{i}^{e+1}$ is generated for the deleted graph. Future queries operate only on $I_{e+1}$ and $U_{e+1}$. Therefore, the deleted graph cannot be returned or tested as an active record after refresh. Any attempt to link future query tokens to stale deleted-graph tokens reduces to distinguishing independently generated epoch-e+1 PRF outputs from random strings plus the explicitly leaked refresh-size metadata.    □

### 6.7. Query and Result Privacy

**Proposition 1** 
(Query-view simulation)**.** *For a query (Q,τ) in epoch e, the server view*

(58) $${\mathrm{View}}_{\mathrm{qry}}\left(Q,\tau ,e\right)=\left(T_{Q}^{e},{\mathrm{path}}_{e}\left(Q\right),{\mathrm{updscan}}_{e}\left(Q\right),{\mathrm{Cand}}_{e}\left(Q,\tau \right),R_{e}\left(Q,\tau \right)\right)$$

*is simulatable from $L_{\mathrm{qry}}\left(Q,\tau ,e\right)$ and cryptographic sizes.*

**Proof.**  The simulator samples pseudo-random query labels for $T_{Q}^{e}$, creates a traversal trace with the leaked path length and node identifiers, samples update buffer checks according to ${\mathrm{updscan}}_{e}\left(Q\right)$, and returns the leaked number of ciphertext records. The plaintext branch labels in *Q* are used only inside PRF inputs and masked count vectors. By Lemmas 1 and 2, replacing these objects with simulated labels and ciphertexts is computationally indistinguishable. The non-hidden parts are exactly traversal pattern, update buffer checks, candidate count, and result size.    □

### 6.8. Re-Randomization Security

**Proposition 2** 
(Cross-epoch unlinkability)**.** *For any active branch occurrence (b,i,c) refreshed from epoch e to e+1, the occurrence handle pair*

(59) $$\left(\eta_{b,i,c}^{e},\eta_{b,i,c}^{e+1}\right)=\left(F_{K_{O}^{e}}(\theta_{b}^{e}\;\|\;h_{i}^{e}\;\|\;c\;\|\;r_{i}),F_{K_{O}^{e+1}}(\theta_{b}^{e+1}\;\|\;h_{i}^{e+1}\;\|\;c\;\|\;r_{i})\right)$$

*is computationally unlinkable across epochs except through $L_{\mathrm{rr}}$ and workload metadata. The cloud does not receive the pair $\left(h_{i}^{e},h_{i}^{e+1}\right)$ during full shuffle refresh. For any PPT linking adversary A,*

(60) $${\mathrm{Adv}}_{\mathrm{link}}\left(A\right)\leq 2{\mathrm{Adv}}_{F}^{\mathrm{prf}}+\mathrm{Pr}\left[\mathrm{MetaColl}\right]+\mathrm{negl}\left(\lambda \right),$$

*where MetaColl denotes a uniqueness event in leaked metadata such as record size buckets, refresh batch timing, or repeated result size patterns.*

**Proof.**  Epoch keys are derived as independent PRF outputs:

(61) $$K_{O}^{e}=F_{K}\left(\mathrm{epoch}\|\;e\;\|\;O\right),\;K_{O}^{e+1}=F_{K}(\mathrm{epoch}\|\;e+1\;\|\;O).$$

Replacing epoch-*e* occurrence handles with random labels and then replacing epoch-(e+1) occurrence handles with independent random labels changes the adversary’s view only by the PRF advantage. Independent random labels do not encode branch or record equality across epochs. Because the refresh package is shuffled, a successful link must use non-token information such as bucketed record size, batch timing, or repeated query result metadata. These correlations are captured by Pr[MetaColl] and the declared re-randomization leakage.    □

### 6.9. Comparison with Static PPGSQ-Style Indexing

In a static PPGSQ-style index, a branch occurrence keeps a stable encrypted identifier. Abstractly, if the same branch occurrence appears in two time windows, the server may observe

(62) $$\eta_{b,i,c}^{e}=\eta_{b,i,c}^{e+1}\;\mathrm{or}\;{\mathrm{path}}_{e}\left(Q\right)\approx {\mathrm{path}}_{e+1}\left(Q\right),$$

which enables longitudinal profiling. Inserted branches can be tested against old query identifiers if update labels reuse the same branch namespace, and deleted branches may remain observable through stale index nodes until a full rebuild is performed.

DFB-PPGSQ changes these equalities into epoch-local relations:

(63) $$\mathrm{Pr}\left[\eta_{b,i,c}^{e}=\eta_{b,i,c}^{e+1}\right]\leq 2^{-\lambda },\;{\mathrm{Act}}_{e+1}=\left({\mathrm{Act}}_{e}\cup {\mathrm{Ins}}_{e}\cup {\mathrm{Upd}}_{e}\right)\setminus D_{e}.$$

Thus, active records are re-tokenized, update records use one-time labels, deleted records are removed from the next searchable state, and the cloud does not receive a pairwise old-to-new refresh map. The price is visible update timing, buffer scans, bucketed refresh sizes, and epoch-maintenance leakage, while the gain is that temporal branch and handle equality are no longer stable server-side identifiers.

## 7. Experimental Evaluation

This section reports a reproducible prototype evaluation of DFB-PPGSQ. The goal is to test whether the proposed dynamic mechanisms preserve the main branch-filtering advantage of PPGSQ-style search while avoiding full index rebuild after ordinary updates. It implements protocol-relevant operations in Python: branch extraction, HMAC-SHA256 token generation, feature token matching, one-time update labels, branch count lower-bound filtering, feature-only dynamic SSE lookup, update buffering, tombstone filtering, and shuffle-based epoch re-randomization.

The evaluation is organized around three contribution-oriented questions: whether dynamic updates avoid immediate full-index rebuilds, whether the protected branch tree preserves the pruning advantage of static PPGSQ-style search, and whether epoch re-randomization reduces cross-epoch linkability while exposing the expected maintenance tradeoff.

### 7.1. Prototype Implementation and Workloads

The evaluation was executed on Windows 11 with Python 3.14.2 on an Intel64 Family 6 Model 189 processor. Every plotted point reports the mean over five independent random seeds, and the error bars show the standard deviation across these seeds. The generated workload models a dynamic sensor topology graph database. Each default mesh graph contains 8–22 vertices. Vertices receive assigned sensor or device labels such as temperature, pressure, gateway, camera, and alarm. Edges encode communication, control, backup, or event relations. A ring backbone keeps each graph connected, and additional sparse edges emulate redundant sensor links or event dependencies. To strengthen the sensor-oriented evaluation, we also add industrial CPS and campus IoT stress profiles with larger graphs and richer device vocabularies, motivated by the dynamic dependency and security requirements observed in sensor network studies [1,2], as shown in Table 3.

**Protocol scope vs. engineering completeness.** The current Python prototype is intentionally scoped to the core protocol logic that directly affects the security and filtering performance claims: branch extraction, HMAC-SHA256 based PRF/token generation, masked count vectors, update buffers, deletion tombstones, tree node aggregate computation, and epoch refresh. However, several components required for a deployable system are deliberately omitted because they are standard engineering layers that do not affect the protocol-level security or filtering latency measurements reported in this paper. These omissions include: (i) authenticated encryption for graph records (the symbolic ${\mathrm{Enc}}_{K}\left(G_{i}\right)$ is used instead of a concrete scheme such as AES-GCM); (ii) persistent client-side storage of epoch keys, logical ID maps, graph nonces, and update counters with rollback protection; (iii) crash recovery mechanisms for partially applied epoch refreshes; and (iv) an exact graph edit distance (GED) refinement engine executed on the client after decryption of returned candidates. Treating these as orthogonal allows us to isolate the contributions of the dynamic structured encryption design from implementation-dependent engineering choices and to focus the evaluation on the protocol-level tradeoffs.

The dynamic workload is denoted by

(64) $$W=\{{\mathrm{op}}_{1},{\mathrm{op}}_{2},\ldots ,{\mathrm{op}}_{T}\},$$

where ${\mathrm{op}}_{t}\in \left\{\mathrm{Insert},\mathrm{Delete},\mathrm{UpdateLabel},\mathrm{Search},\mathrm{ReRand}\right\}$. A workload profile is represented as

(65) $$\pi =(p_{\mathrm{ins}},p_{\mathrm{del}},p_{\mathrm{lab}},p_{\mathrm{qry}},E),$$

where *E* is the epoch length. Insertions generate new sensor topology graphs, deletions insert epoch-specific tombstone handles, and label updates perturb local vertex or edge labels to regenerate only the affected branches. Queries are generated by perturbing existing graphs by one to three local label changes.

### 7.2. Baselines

The comparison includes both graph similarity baselines and dynamic encrypted search baselines. Table 4 summarizes their roles. Static PPGSQ1 performs full branch lower-bound scanning. Static PPGSQ2 uses the same size and branch count filtering principle as a branch–tree baseline, but does not include dynamic privacy. DSSE-branch represents a feature-only dynamic searchable encryption service that retrieves graphs sharing authorized sensor labels but does not maintain a graph similarity branch tree. Dynamic rebuild rebuilds the whole protected index after each update. Finally, the no-re-randomization baseline keeps stable branch identifiers, and is used only for linkability testing.

### 7.3. Metrics

For a query set Q, average query latency is

(66) $${\bar{T}}_{q}=\frac{1}{\left|Q\right|}\sum_{Q\in Q}T_{q}\left(Q\right).$$

For an update stream U, average update latency is

(67) $${\bar{T}}_{u}=\frac{1}{\left|U\right|}\sum_{\mathrm{op}\in U}T_{u}\left(\mathrm{op}\right).$$

The pruning ratio for query *Q* is

(68) $$\mathrm{PR}\left(Q\right)=1-\frac{\left|\mathrm{Cand}(Q)\right|}{\left|G\right|}.$$

The false positive candidate ratio after local refinement is

(69) $$\mathrm{FPR}\left(Q\right)=\frac{|\mathrm{Cand}\left(Q\right)\setminus R^{★}\left(Q\right)|}{\left|\mathrm{Cand}(Q)\right|},$$

where $R^{★}\left(Q\right)$ is the exact result set under the graph edit distance threshold. Linkability attack success is measured as

(70) $$\mathrm{ASR}=\frac{\#\mathrm{correctly}\;\mathrm{linked}\;\mathrm{epoch}-\mathrm{crossing}\;\mathrm{tokens}}{\#\mathrm{tested}\;\mathrm{token}\;\mathrm{pairs}}.$$

Storage and communication overhead are not optimized in this Python prototype, and are not used for the main comparison. The reported metrics are query latency, update latency, epoch refresh maintenance cost, candidate pruning ratio, and linkability attack success. The stress profile experiment additionally reports a metadata-assisted linkability attack that combines branch label length, local degree, and coarse branch string length buckets. Latency consists of wall clock measurements in milliseconds.

### 7.4. Query Scalability

**Figure 3:**
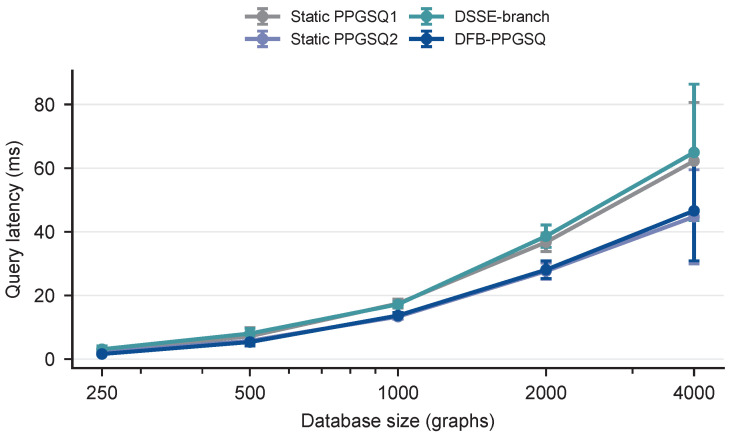
Query latency under increasing graph database scale. The legend has been moved outside the plotting area; points show mean values over five random seeds and error bars show standard deviations.

The first experiment measures query latency as the graph database grows from 250 to 4000 graphs. Figure 3 shows that DFB-PPGSQ remains close to the static branch–tree baseline while outperforming full scanning and feature-only DSSE at larger scales. At 4000 graphs, DFB-PPGSQ required 46.60 ms on average, compared with 44.72 ms for Static PPGSQ2, 62.22 ms for Static PPGSQ1, and 64.95 ms for DSSE-branch. Thus, the dynamic mechanisms add about 4.2% query overhead relative to the static branch–tree baseline at the largest tested scale while avoiding a return to linear scanning of the full database.

### 7.5. Dynamic Update Cost

**Figure 4:**
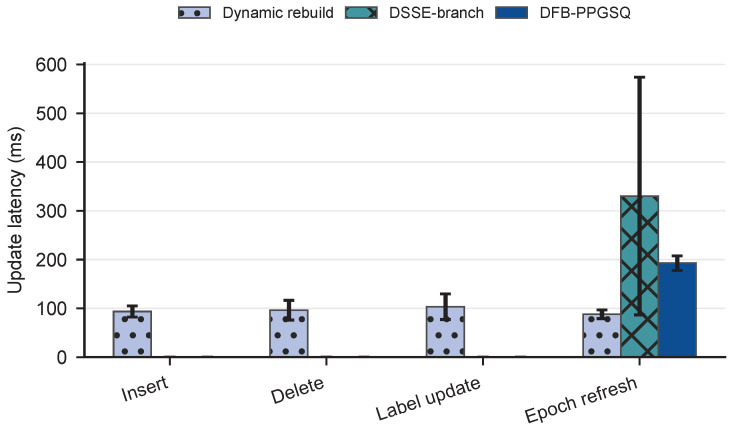
Dynamic update latency for insertion, deletion, label update, and epoch refresh. The legend has been separated from the bars; bars show mean values over five random seeds and error bars show standard deviations.

The second experiment separates ordinary updates from epoch maintenance. Figure 4 shows that immediate rebuild is expensive for insertions, deletions, and label updates because the whole protected index is reconstructed after every operation. In contrast, DFB-PPGSQ appends insertions and label updates to the update buffer and stores deletions as tombstones. In the measured prototype, insertion and label update cost 0.091 ms and 0.092 ms on average, respectively, while delete cost 0.007 ms. Epoch refresh remains expensive because it rebuilds and re-randomizes the active index. Its mean cost was 192.73 ms, which is expected because DFB-PPGSQ moves rebuild cost from every update to scheduled epoch boundaries.

### 7.6. Re-Randomization Tradeoff

**Figure 5:**
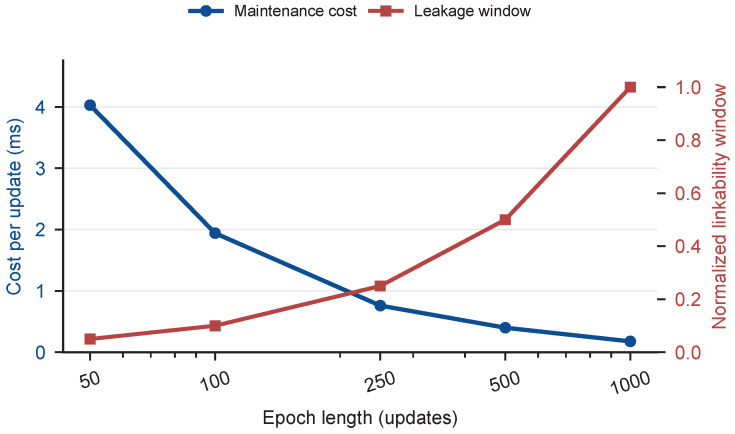
Epoch-length tradeoff between amortized re-randomization maintenance cost and normalized linkability window. The dual-axis legend has been placed above the plot to avoid crossing plotted lines or labels.

The third experiment varies the epoch length. A shorter epoch reduces the time window in which tombstone and update buffer traces remain linkable, but increases the amortized maintenance cost. Figure 5 shows this tradeoff. The measured maintenance cost falls from 4.03 ms per update at an epoch length of 50 updates to 0.177 ms per update at 1000 updates. The normalized linkability window grows in the opposite direction, since a longer epoch leaves stable within-epoch metadata visible for a longer period.

### 7.7. Pruning Ability

**Figure 6:**
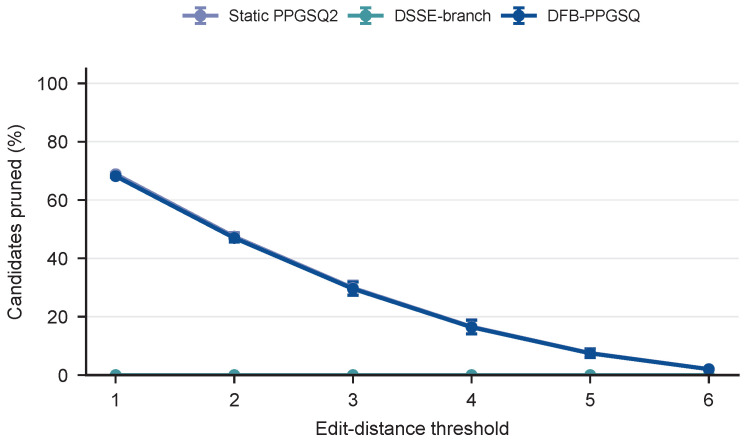
Candidate pruning ratio under different edit distance thresholds. The legend has been placed outside the plotting area; points show mean values over five random seeds and error bars show standard deviations.

The fourth experiment evaluates whether dynamic privacy weakens branch-based pruning. Figure 6 reports the fraction of graphs removed before refinement. DFB-PPGSQ closely tracks the static branch–tree baseline because it preserves the same lower-bound filtering structure and adds only update buffer and tombstone checks. At τ=1, Static PPGSQ2 pruned 68.9% of candidates, while DFB-PPGSQ pruned 68.2%. At τ=6, the pruning ratios were 2.0% and 2.0%, respectively. The feature-only DSSE baseline pruned almost no graphs in this generated sensor workload because most graphs share at least one coarse sensor label; this illustrates why dynamic keyword-style search is not a substitute for graph similarity filtering.

### 7.8. Sensor Network Stress Workloads

**Figure 7:**
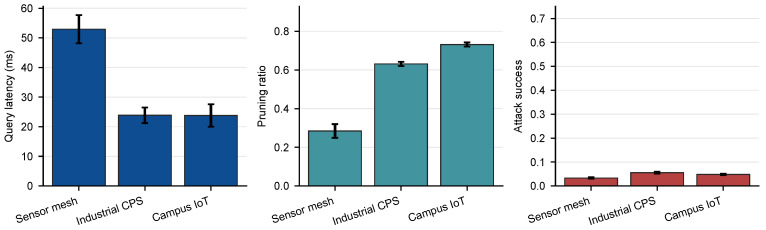
Sensor network stress workloads. The three panels report DFB-PPGSQ query latency, candidate pruning ratio, and metadata-assisted cross-epoch linkability attack success over mesh, industrial CPS, and campus IoT graph profiles, with adjusted panel spacing for readability. Bars show mean values over five random seeds and error bars show standard deviations.

The fifth experiment tests whether the same implementation remains stable under more heterogeneous sensor network profiles. Figure 7 reports three workloads. The default sensor mesh profile uses 4000 graphs with 14.98 vertices per graph on average. The industrial CPS and campus IoT profiles use 1500 larger graphs with 40.05 and 56.16 vertices per graph on average, respectively, and include richer device and communication labels. DFB-PPGSQ required 52.92 ms on the 4000-graph mesh profile, 23.86 ms on the industrial CPS profile, and 23.80 ms on the campus IoT profile. The two profiles with larger graphs also produced stronger pruning, which is because their richer labels and higher local degree make branch summaries more selective. The, respective pruning ratios were 63.2% and 73.2%. Under a stronger metadata-assisted cross-epoch attacker using label length, degree, and coarse branch-string buckets, the measured attack success rates for the three profiles were 3.3%, 5.6%, and 4.8%, respectively. These results do not replace evaluation on public IoT traces, but give a stronger stress test than a single small synthetic topology.

### 7.9. Resilience to Leakage

**Figure 8:**
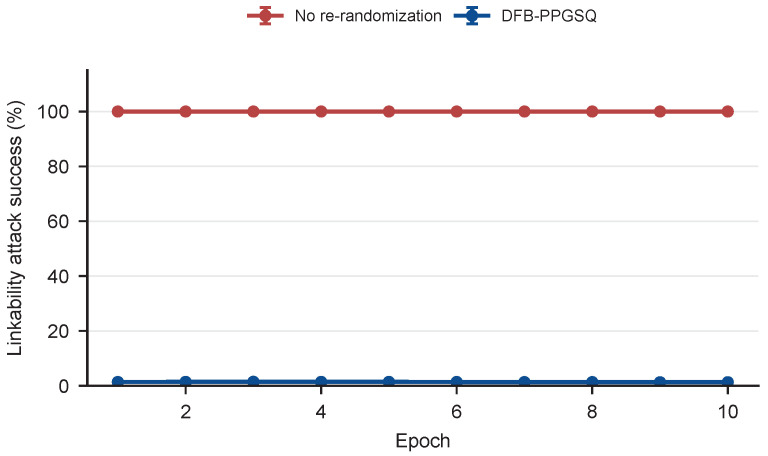
Linkability attack success rate across epochs with and without periodic re-randomization. The legend and vertical range have been adjusted so that the no-re-randomization curve is not obscured; points show mean values over five random seeds and error bars show standard deviations.

The sixth experiment evaluates temporal linkability under a simple attacker. For the no-re-randomization baseline, the attacker links occurrence handles across epochs by deterministic equality. For DFB-PPGSQ, equality no longer works after epoch refresh, and the attacker falls back to coarse bucket matching based on branch label length and local degree. Figure 8 shows that stable identifiers make occurrence–handle linkage trivial in the baseline, whereas DFB-PPGSQ reduces the measured bucket-matching success rate to approximately 1.4–1.5%. While this experiment does not prove resistance to all leakage abuse attacks, it demonstrates that refreshing the epoch key removes the strongest equality signal under the declared leakage model, leaving only coarse metadata leakage.

### 7.10. Prototype Scope and Deployment Limitations

The prototype is intended to evaluate the protocol-relevant filtering path, not to be a complete production encrypted graph database. It implements branch extraction, HMAC-SHA256 based PRF/token simulation, masked count vectors, update buffers, tombstones, and epoch refresh. A deployable system should instantiate ${\mathrm{Enc}}_{K}\left(G_{i}\right)$ with authenticated encryption, ensure persistence of the client’s epoch state and counters with rollback protection, provide crash recovery for partially applied refreshes, and use constant-time cryptographic library implementations. These engineering requirements are outside the current prototype, and are not included in the measured latency results.

The reported query latency measures server-side encrypted filtering before local exact refinement. In an end-to-end deployment, the client must decrypt returned candidates and run an exact or approximate graph edit distance refinement engine. The local refinement cost depends on the candidate count, query size, graph size, and chosen GED implementation. Thus, the pruning ratio experiment reports the main cost driver for refinement, and should not be read as a complete end-to-end latency claim.

The storage and communication overheads are also explicit. Let $\bar{B}$ be the average number of branch occurrences per graph, *m* the count vector length, λ the token length, and $|U_{e}\left|,\right|D_{e}|$ the current buffer and tombstone sizes. The protected server-side storage has the following order:

(71) $$O\;\left(\sum_{i}|\mathrm{Enc}\left(G_{i}\right)|+N\bar{B}\lambda +Nm\log p+|U_{e}|\left(\bar{B}\lambda +m\log p\right)+\left|D_{e}\right|\lambda \right).$$

A query token contains current-epoch feature tokens, protected query counts, and update buffer probes, giving communication on the order of

(72) $$O\;\left(|B\left(Q\right)|\lambda +m\log p+\left|B\left(Q\right)|\;\right|U_{e}|\lambda \right),$$

while a full refresh uploads a shuffled active-state package of size

(73) $$O\;\left(|M_{e}|\left(\bar{B}\lambda +m\log p\right)\right).$$

These expressions explain why epoch length, update buffer growth, and vector length must be tuned for a target sensor cloud deployment.

The leakage resilience experiments test equality linkage removal and a metadata-assisted bucket attacker; however, they do not cover stronger statistical or machine learning attackers that combine traversal paths, repeated result sizes, public IoT graph distributions, update timing, and refresh sizes. The present construction also does not hide volume, result size, update time, or tree traversal. Deployments that require stronger leakage profiles should add padding, batched updates, dummy records, fixed-rate refresh, oblivious traversal, or ORAM-like access. The cost of these additions is not measured in the current prototype.

Finally, the threat model used in this paper is honest-but-curious. It does not verify whether the cloud returned all and only correct candidates; a malicious or compromised server could omit results, replay stale nodes, or return malformed ciphertexts unless the system is augmented with authenticated data structures, verifiable search proofs, or consistency audits. Supporting malicious-server integrity, multi-owner authorization, edge device energy profiling, public IoT traces, and explicit edge-insertion/edge-deletion implementations are important directions for future work.

## 8. Conclusions

This paper proposes DFB-PPGSQ, a dynamic forward-private and epochal backward-private graph similarity matching query scheme over encrypted graph databases. The scheme targets a realistic sensor cloud setting where graphs are not static and need to be inserted, deleted, relabeled, and periodically maintained.

DFB-PPGSQ combines branch-based lower-bound filtering with dynamic structured encryption. It introduces epoch-local feature tokens, per-record occurrence handles, update buffers, deletion tombstones, and shuffle-based periodic re-randomization. These mechanisms preserve efficient graph similarity filtering while reducing temporal leakage from dynamic updates. The security analysis shows how the scheme achieves forward privacy for future insertions and label updates and epochal backward privacy for deleted graphs after compaction under an explicit leakage model. The prototype evaluation supports the design goal under which ordinary updates avoid immediate full rebuild, query latency remains close to the static branch–tree baseline, stress workloads remain practical on heterogeneous sensor topologies, and epoch refresh removes stable cross-epoch token equality.

Future work will extend the prototype to public IoT and cyber–physical graph traces, optimize constant-time cryptographic implementations, measure client-side energy and exact refinement costs, and investigate verifiable multi-user and stronger leakage-resistant encrypted graph services.

## Figures and Tables

**Table 1:** Functional and privacy comparison with related encrypted graph querying schemes.

Scheme	Mechanism Coverage						Residual Risk
	**Sim.**	**Dyn.**	**Label**	**Re-Rand.**	**Fwd.**	**Bwd.**	
PGSim [3]	•	×	×	×	×	×	stable index leakage
PrigSim [4]	•	×	×	×	×	×	full-database traversal leakage
PPGSQ [5]	•	×	×	∘	×	×	static branch-token linkage
GraphShield [25]	×	•	∘	∘	•	∘	not designed for similarity filtering
Dynamic SSE index [17,21,22]	∘	•	∘	∘	•	∘	weak graph-structure pruning
DFB-PPGSQ	•	•	•	•	•	∘	same-epoch tombstone leakage

•: directly supported; ∘: bounded, indirect, or assumption-dependent support; ×: outside the current mechanism. Sim.: graph similarity query; Dyn.: insertion/deletion; Label: vertex/edge label updates; Re-Rand.: periodic re-randomization or compaction.

**Table 2:** Notation used in the DFB-PPGSQ construction and security analysis.

Symbol	Description
λ	Security parameter
$G^{t}$	Dynamic graph database at time *t*
$G_{i}$	The *i*-th data graph
*Q*	Query graph
τ	Graph edit distance threshold
B(G)	Branch multiset extracted from graph *G*
*b*	A branch feature
*e*	Current epoch
$K_{e}$	Epoch key
$\theta_{b}^{e}$	Epoch-local query-matchable feature token of branch *b*
$\eta_{b,i,c}^{e}$	Per-record occurrence handle for the *c*-th occurrence of branch *b* in graph $G_{i}$
$I_{e}$	Encrypted branch–tree index in epoch *e*
$U_{e}$	Encrypted update buffer in epoch *e*
$D_{e}$	Deletion tombstone set in epoch *e*
LBED	Branch-based lower bound of edit distance
op	Dynamic operation type

**Table 3:** Prototype workload and reproducibility settings.

Component	Setting	Range/Value	Purpose
Graph database size	generated sensor topology graphs	250, 500, 1000, 2000, 4000 graphs	query scalability
Graph structure	connected sparse graph	8–22 vertices; ring backbone plus random extra edges	sensor network topology model
Stress profiles	mesh, industrial CPS, campus IoT	1500–4000 graphs; mean 15–56 vertices	robustness to heterogeneous sensor networks
Dynamic operations	insert, delete, label update, epoch refresh	five random seeds: 1701–1705	update and maintenance cost
Query threshold	graph edit distance threshold proxy	τ∈{1,2,3,4,5,6}	pruning sensitivity
Epoch length	refresh interval	50, 100, 250, 500, 1000 updates	privacy–efficiency tradeoff

**Table 4:** Experimental baselines.

Method	Mechanism Profile			Purpose
	**Query**	**Update**	**Privacy**	
Static PPGSQ1-style	linear branch scan	rebuild only	content privacy	query-cost lower baseline
Static PPGSQ2-style	branch-tree pruning	rebuild only	content privacy	strongest static pruning baseline
Rebuild-after-update	branch-tree pruning	full rebuild	freshness	maintenance-cost upper baseline
DSSE-branch	feature-only lookup	dynamic update	forward privacy	cost of losing branch-tree pruning
No re-randomization	dynamic branch-tree	buffered update	partial privacy	value of epoch refresh
DFB-PPGSQ	dynamic branch-tree	buffer and compaction	forward/backward privacy	proposed construction

## Data Availability

The prototype script, generated workload settings, measured CSV files, and figure-generation outputs are included with this manuscript package under scripts/, source_data/, and figures/. No human-subject data or third-party restricted datasets are used in the reported prototype evaluation.

## Abbreviations

The following abbreviations are used in this manuscript:

DFB-PPGSQ	Dynamic Forward/Backward-Private Privacy-Preserving Graph Similarity Query
DSSE	Dynamic Searchable Symmetric Encryption
GED	Graph Edit Distance
HMAC	Hash-Based Message Authentication Code
IoT	Internet of Things
PPGSQ	Privacy-Preserving Graph Similarity Query
SSE	Searchable Symmetric Encryption
